# Performance of artificial intelligence models designed as adjuncts for determining working length and apical landmark assessment in endodontic procedures: a systematic review

**DOI:** 10.3389/fdmed.2026.1783828

**Published:** 2026-03-06

**Authors:** Sanjeev B. Khanagar, Majed Alharthi, Adel Asery, Muhammad Alalweet, Abdullah Aldobiyan, Meshal Alhayf, Ahmed Binobaid

**Affiliations:** 1 Preventive Dental Science Department, College of Dentistry, King Saud bin Abdulaziz University for Health Sciences, Riyadh, Saudi Arabia; 2 King Abdullah International Medical Research Centre, Riyadh, Saudi Arabia; 3 Ministry of the National Guard Health Affairs, Riyadh, Saudi Arabia; 4 College of Dentistry, King Saud bin Abdulaziz University for Health Sciences, Riyadh, Saudi Arabia; 5 Department of Restorative and Prosthetic Dental Sciences, College of Dentistry, King Saud bin Abdulaziz University for Health Sciences, Riyadh, Saudi Arabia

**Keywords:** apical foramen, artificial intelligence, automated, endodontics, machine learning, root apex, root length, working length

## Abstract

**Background:**

The success of endodontic procedure depends on the precise determination of the working length (WL), which is measured from a coronal reference point to the apical constriction of the root canal. Accurate WL measurement ensures thorough debridement, effective disinfection, and optimal obturation, thereby preventing complications such as postoperative pain, over instrumentation, and persistent infection. Given the emerging use of artificial intelligence (AI)-based computational models in endodontics, this systematic review aimed to evaluate the performance of AI models developed to assist in determining WL length and identifying related apical landmarks in endodontic procedures.

**Methods:**

A comprehensive search of PubMed, Scopus, Embase, Cochrane Library, Web of Science, and Google Scholar was conducted from January 1, 2000, to July 31, 2025. Eligible studies included those evaluating machine learning or neural network–based models for WL assessment. Methodological quality was appraised using QUADAS-2, with explicit differentiation between risk of bias (internal validity) and applicability (external validity). The certainty of evidence was assessed using the GRADE approach.

**Results:**

Six studies met the eligibility criteria. Substantial heterogeneity was observed in algorithm types (e.g., artificial neural networks, ensemble machine learning models), input modalities (radiographic vs. impedance-based), reference standards, and validation strategies. Most studies relied on retrospective or *in vitro* datasets with internal validation only; no study reported prospective, real-world external validation. The QUADAS-2 assessment identified concerns related to patient selection and applicability, particularly in studies using extracted teeth or experimental datasets. According to GRADE, the overall certainty of the evidence was low. Reported performance metrics varied, with sensitivity ranging from 0.85–1.00, specificity from 0.50–1.00, and accuracy from 0.70–0.95. However, comparisons across studies were limited by inconsistent outcome definitions, the absence of standardized clinical error thresholds (e.g., ±0.5 mm), and infrequent reporting of confidence intervals.

**Conclusion:**

AI-based models show preliminary and investigational potential as adjunctive tools for WL determination. However, the current evidence is limited by methodological heterogeneity, reliance on non-clinical datasets, and a lack of external validation. None of the included studies provide high-certainty evidence from prospective, real-world clinical trials. Therefore, AI systems should currently be considered adjunctive and experimental rather than clinically established. Future research should prioritize prospective, multicenter clinical validation, standardized outcome definitions, and transparent reporting to enhance generalizability and clinical applicability.

**Systematic Review Registration:**

https://www.crd.york.ac.uk/PROSPERO/view/CRD420251231561. PROSPERO CRD420251231561.

## Introduction

Oral diseases rank among the most prevalent health issues globally, creating considerable health and economic challenges while substantially reducing the quality of life for those impacted (1). Dental caries is the most prevalent and impactful oral disease globally, representing a major public health concern by affecting billions of people and resulting in high treatment costs (1, 2). The global prevalence of dental caries is particularly alarming, affecting nearly 2 billion individuals and increasing the risk of developing periapical lesions due to untreated or poorly managed infections (3). The consequences of chronic, untreated oral diseases can be quite serious, encompassing ongoing pain, sepsis, diminished quality of life, absenteeism from school, disruption of family schedules, and lowered work efficiency (1).

Endodontic treatment, commonly known as root canal therapy, is a vital dental procedure aimed at preserving teeth with irreversibly inflamed or necrotic pulps. This is achieved by removing infected tissue, disinfecting the root canal system, and sealing it to prevent reinfection (4). The success of this procedure depends on the precise determination of the working length (WL), which is from a coronal reference point to the apical constriction of the root canal (5). Accurate WL measurement ensures thorough debridement, effective disinfection, and optimal obturation, thereby preventing complications such as postoperative pain, over instrumentation, inadequate removal of bacteria causing persistent infection (6).

Conventionally, WL determination has relied on techniques such as radiographic methods (e.g., paralleling or bisecting-angle radiographs) and electronic apex locators (EALs), which measure resistance or impedance changes at the apical constriction (7). While radiographs remain a gold standard, they present challenges, including radiation, superimposition, and subjectivity in interpretation (8). While EALs, demonstrate high clinical reliability, certain anatomical and procedural factors (e.g., open apices or complex canal anatomy) may introduce interpretative challenges (9). Emerging Artificial Intelligence (AI)-based approaches are therefore being investigated as adjunctive tools to support interpretation and standardization, rather than as replacements for established working length determination methods.

Recent advancements in AI have revolutionized diagnostics and treatment planning in dentistry, particularly in image analysis, caries detection, and the identification of periapical lesions (10, 11). In endodontics, AI-powered systems, can analyze radiographic data to automate the identification of anatomical landmarks and measure root canal dimensions (12). These AI-driven models have shown exceptional performance in detecting, segmenting, and classifying periapical lesions (13, 14). AI technologies can minimize human error, standardize assessments, and reduce procedural time, thereby enhancing clinical outcomes. However, challenges persist, including the need for large, diverse training datasets and validation in real-world clinical settings. In this review, WL determination refers to estimating the distance from a coronal reference point to the apical constriction or minor apical foramen, which represents the clinically optimal endpoint for cleaning and obturation. However, it is acknowledged that some studies evaluate related anatomical or procedural parameters, such as minor apical foramen localization, canal length estimation, apical extent of instrumentation, or post-obturation filling length that indirectly contribute to or influence WL determination. Given the close anatomical and clinical interrelationship of these parameters, such studies were included when their AI models directly assisted in estimating or approximating the apical termination point used in WL assessment. Hence the aim of this review was to evaluate the performance of AI models developed to assist in determining WL length and identifying related apical landmarks in endodontic procedures.

## Materials and methods

This systematic review was carried out in compliance with the diagnostic test accuracy standards specified in the Preferred Reporting Items for Systematic Reviews and Meta-Analyses Extension for Diagnostic Test Accuracy (PRISMA-DTA) (15) (Supplementary Table S1).

The protocol for this review was registered with PROSPERO under the ID number CRD420251231561. The literature search was structured using the PICO framework (Population, Intervention, Comparison, and Outcome). The research question was: What is the performance of AI models developed to determine the working length in endodontics? The population included patients who underwent evaluations or interventions for pulpal and periapical conditions or root canal treatment. The intervention comprised AI-based models developed to determine the working length in endodontics. The outcomes considered included measurable or predictive metrics such as accuracy, sensitivity, specificity, precision, F-measure, positive predictive value (PPV), and negative predictive value (NPV).

### Search strategy

Several reputable databases, including PubMed, Scopus, Embase, Cochrane, Web of Science, and Google Scholar were used to conduct a comprehensive digital search for relevant data. Our extensive search covered the period from January 1, 2000, to July 31, 2025.

To search for articles in electronic databases several key terms were employed including artificial intelligence, convolutional neural network, automated, machine learning, deep learning, apical foramen, root apex, working length, root length, medical image analysis, endodontics, periapical radiograph, apical foramen, apex locator, determination, assessment and estimation. Additionally, Boolean operators (AND, OR) were applied and only studies published in English were included. To supplement our electronic search, we also manually reviewed pertinent research publications and their citations including examining the reference lists of previously collected articles in the college library. The search was conducted by two distinct authors who were specifically trained for this task.

The search strategy used for searching the articles in the electronic databases comprised of key terms and Boolean operators AND/OR. It comprised of Endodontics, root canal therapy, working length, root length, root apex, artificial intelligence, machine learning, automation, dental radiographs, medical image analysis, periapical radiographs, apical foramen, apex locator, deep learning, convolutional neural networks, image segmentation, root canal, determination, assessment, and estimation. The search process was conducted independently by two reviewers, with discrepancies resolved through discussion. Complete search strategies for all databases are provided in the Supplementary Table S2 to ensure reproducibility. Google Scholar was searched using a simplified combination of key terms: “artificial intelligence” AND “working length” AND “endodontics.” Results were sorted by relevance, and the first 200 results (corresponding to the first 10 pages) were manually screened. Duplicate records were removed prior to screening. No additional filters were applied beyond the predefined date range and language criteria.

### Eligibility criteria

Following eligibility criteria were set for inclusion: (a) Articles must represent original contributions focused on AI; (b) they should incorporate quantitative data for analysis; (c) they must provide clear references to the data that facilitate the assessment of AI-based models and (d) Studies evaluating AI-based identification of anatomical landmarks or procedural parameters that directly influence WL determination (e.g., minor apical foramen detection, canal length estimation, or apical extent measurement) were also included, provided that the outcomes contributed to clinical WL assessment. Furthermore, there were no restrictions placed on the study design for inclusion in this review. Excluded from this review were articles that did not focus on AI innovation, unpublished conference papers or those that were only accessible online, unpublished works, articles without full-text availability, pilot studies (a pilot study was operationally defined as a preliminary investigation explicitly described by the authors as a pilot, feasibility, or proof-of-concept study, primarily intended to test study procedures rather than to assess diagnostic performance.), and those not in English.

### Study selection

Two researchers, S.B.K. and A.O., carried out the search process independently. The initial search identified 154 records (152 from electronic databases and 2 from manual searching). After removing 84 duplicates, 70 records were screened by title and abstract. Of these, 64 were excluded, and 6 studies were assessed for full-text eligibility and subsequently included in the qualitative synthesis.

### Data extraction

**Figure 1:**
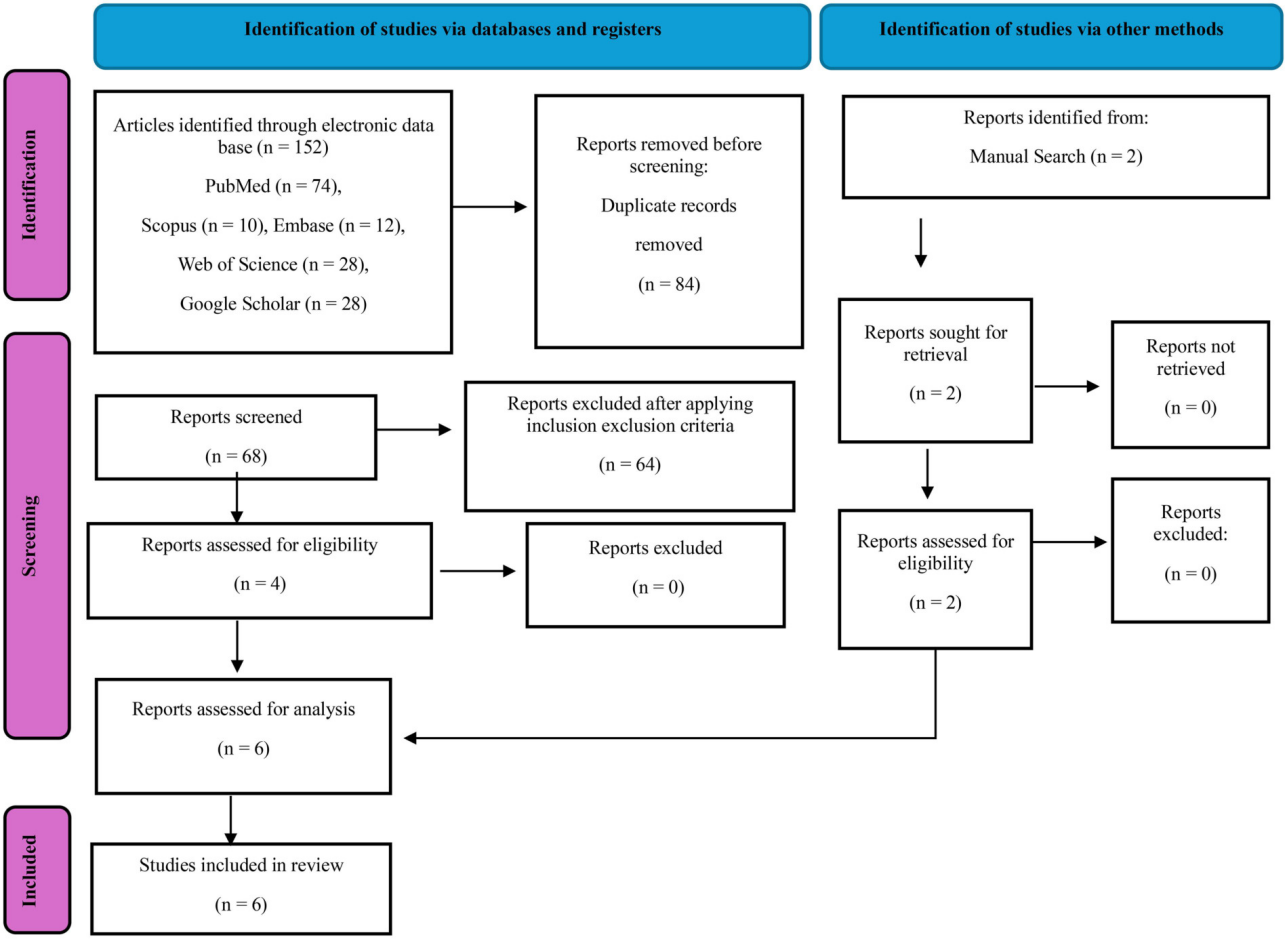
PRISMA 2020 flow diagram for new systematic reviews which included searches of databases, registers and other sources.

To ensure objectivity, the names of the journals and authors were removed, allowing two independent reviewers (M.A.A and M.A.), to assess the articles. Relevant information from the chosen papers was meticulously extracted and entered a Microsoft Excel spreadsheet, which contained details regarding authors, publication dates, research aims, types of AI algorithms employed, and the data utilized for model training, validation, and testing. Furthermore, the outcomes, findings, and recommendations from the studies were also recorded. Disputes that arose during this process were resolved after consulting with a qualified author (A.O). Consequently, a total of 6 articles that were meticulously selected and analyzed in this review, as illustrated in Figure 1.

The studies were assessed for quality through the QUADAS-2 framework (16), which scrutinized various elements of research design and reporting, such as patient selection, index test, reference standard, flow, and timing. This evaluation sought to ascertain the applicability of the findings across diverse clinical environments and patient demographics while pinpointing possible sources of bias. The reliability of the studies incorporated in this systematic review was appraised using the Grading of Recommendations Assessment Development and Evaluation (GRADE) (17). Two reviewers (M.A.A and A.A.D), demonstrated a significant level of agreement, achieving an 82% concordance as indicated by Cohen's kappa.

## Results

Following a comprehensive examination of six articles, qualitative data were gathered. These articles were published from 2012 to 2024. The included studies were categorized into four subgroups according to their primary outcome measures. Direct determination of WL, localization of minor apical foramen, canal length estimation, post-treatment assessment of apical extent or root filling length (18–23). Although these outcomes are not identical, all contribute to the clinical process of WL determination or its verification. Due to methodological heterogeneity and limited sample sizes within each subgroup, a pooled analysis was not performed.

### Qualitative data of the studies

AI has been utilized to ascertain the working length; nevertheless, the data gathered from the studies encompassed a wide variety of samples employed to assess the efficacy of the AI models (18–23). The descriptive information from the studies included is presented in (Table 1).

**Table 1:** Details of the studies that have used AI based models to determine the working length in endodontics.

Serial no	Authors	Year of publication	Study design	Algorithm architecture	Objective of the study	No. of patients’/images/ photographs/ samples for testing	Study factor	Modality	Comparison if any	Evaluation accuracy/average accuracy/ statistical significance	Results (+) effective, (−) non effective (*N*) neutral	Outcomes	Authors suggestions/ conclusions
1	Saghiri M. A. et al. (18)	2011	Comparative study	ANNs	AI-based model for locating the minor apical foramen	50 teeth	Apical foramen	Intra oral radiographs (36 for training 14 for testing)	Two experienced Endodontists	For 93% of the samples, the model determined the location of apical foramen correctly	(+) Effective	ANNs-based model demonstrated good accuracy in detecting the apical foramen	The AI model can serve as a valuable tool for obtaining a secondary opinion, thereby enhancing clinical decision-making.
2	Saghiri M. A et al. (19)	2012	Comparative study	ANNs	AI-based model for determining the working length.	50 teeth	Working length	Intra oral radiographs	Endodontists	AI model demonstrated 96% accuracy in comparison with the experienced endodontists whose accuracy was 76%.	(+) Effective	AI model demonstrated more accuracy in determining the working length in comparison with experienced endodontists who demonstrated an accuracy of 76%	This model proved to be effective in establishing the working length and can act. alternative to electronic apex locators.
3	Qiao, X.et al. (20)	2020	Comparative study	ANNs	To present a multifrequency impedance method based on a neural network for root canal length	21 teeth	Root canal length measurement	Experimental	The dual frequency impedance ratio method	The neural network based multifrequency method exhibits nearly 95%% while the dual frequency impedance ratio method demonstrated no more than 85% in some situations	(+) Effective	The experimental results indicated that the proposed measurements method is relatively robust and can improve the effects of measuring factors on the results	In comparison to the dual frequency impedance ratio method, the suggested approach minimizes the impact of human and environmental factors on the measurement of tooth canal length, thereby improving measurement accuracy and enhancing robustness.
4	Thakur, V.S. et al. (21)	2022	*In vitro* study	ML	To evaluate the dimensions of the apical extent after and during endodontic treatment using a Machine Learning (ML) model to enhance the accuracy of root canal treatment	171 teeth	Apical extent during and after in endodontic treatment	Digital intraoral radiographic imaging	The study compared the performance of different ensemble classifier. Bagged Trees, Boosted Trees, and *Random under Sampling* (RUS) Boosted trees in classifying the apical condition.	The ensemble Bagged Trees model achieved a maximum accuracy of 94.2%, Boosted Trees achieved 91.7% and RUS Boosted trees achieved 90.8%.	(+) Effective	The ensemble Bagged Trees model worked incredibly well can provide a suitable decision support system in endodontics.	The ML models have the potential to enhance treatment outcomes in root canal procedures by providing a reliable decision support system.
5	Herbst, S.R. et al. (22)	2023	Comparative study	ML Logistic regression (logR),	To identify optimal root filling length (RFL) during orthograde root canal treatments using multiple ML models. Random Forest (RF), Support Vector Machine (SVM), Decision Tree (DT), Gradient Boosting Machine (GBM), Extreme Gradient Boosting (XGB)	343 patients, 555 teeth included in the study	Preoperative risk assessment and optimal root filling length	Radiographic evaluation using beam-guiding devices and diagnostic software	Comparison between undergraduate students and postgraduate dentists in achieving optimal RFL; performance comparison of machine learning algorithms	The models’ sensitivity varied from 52.7% for (SVM) to 69.2% for (logR), while specificity ranged from 69.3% for (logR) to 84.9% for (DT). The accuracy of RF (78.6%), XGB (78.9%), and DT (79.6%) was significantly higher compared to (logR) (63.4%), GBM (69.7%), and SVM (66.0%).	(−) non effective	The accuracy of predicting the technical results of a root canal treatment using machine learning algorithms was inadequate.	A preoperative risk assessment is crucial; however, it is insufficient for forecasting RFL through ML algorithms. Future tools ought to concentrate on enhancing the evaluation of risk factors and incorporating clinical parameters.
6	Latke, V. and Narawade, V. (23)	2024	Cross sectional Study	ML	AI-based model to measure the endodontic working length	1,551 sample images	Endodontic working length	Intra oral radiographs	Dental professionals	The system achieved an average accuracy of 96.21% for 10 images and overall average of 86.51% for the 1,551 images	(+) Effective	The results highlight the effectiveness of the image processing pipeline in enhancing diagnostic clarity and accuracy in dental practice. Additionally, it aids clinicians in making well-informed treatment decisions, underscoring its significance in modern dental diagnostics.	The results highlight the effectiveness of the image processing pipeline in enhancing diagnostic clarity and accuracy in dental practice. Additionally, it aids clinicians in making well-informed treatment decisions, underscoring its significance in modern dental diagnostics.

ML, machine learning; ANNs, artificial neural networks.

### Study characteristics

The characteristics of the study included information regarding the authors, the publication year, the objectives of the research, the algorithms utilized for the development of the AI model, the sources of data for training, validation, and testing, the assessment of model accuracy, the findings of the research, and any recommendations provided by the authors.

### Outcome measures

The effectiveness of task execution was assessed using various metrics, including quantifiable or predictive results such as accuracy, sensitivity, specificity, precision, receiver operating characteristic (ROC), and area under the curve (AUC).

### AI model characteristics and methodological features

A structured evaluation of AI model characteristics was conducted to enhance transparency and comparability across studies, revealing substantial heterogeneity in algorithm architecture, input modalities, reference standards, and validation strategies. Two studies employed artificial neural networks (ANNs) for apical foramen localization and working length determination (18, 19), while one study utilized a neural network–based multifrequency impedance model (20); three additional investigations applied classical machine learning approaches, including ensemble classifiers (bagged trees, boosted trees, and RUS boosted trees) as well as logistic regression, support vector machines, random forests, decision trees, gradient boosting machines, and extreme gradient boosting (20–23). Most studies used intraoral periapical radiographs as input data, whereas one relied on impedance-based electrical measurements rather than imaging (20), and image preprocessing procedures were inconsistently reported. Reference standards varied across studies and included expert endodontist assessments, the dual-frequency impedance ratio method, or radiographic consensus, although detailed annotation protocols were often insufficiently described. Sample sizes ranged from 21 teeth to 1,551 radiographic images, and only a minority of studies clearly specified dataset partitioning (e.g., 36 training and 14 testing images) (18); notably, none reported external validation using independent datasets. Validation strategies were predominantly limited to internal validation, thereby restricting generalizability and increasing the risk of overfitting. Given the marked heterogeneity in datasets, model architectures, and validation methods, direct comparisons between classical machine learning and deep learning approaches were not feasible.

### Risk of bias assessment and applicability concern

**Figure 2:**
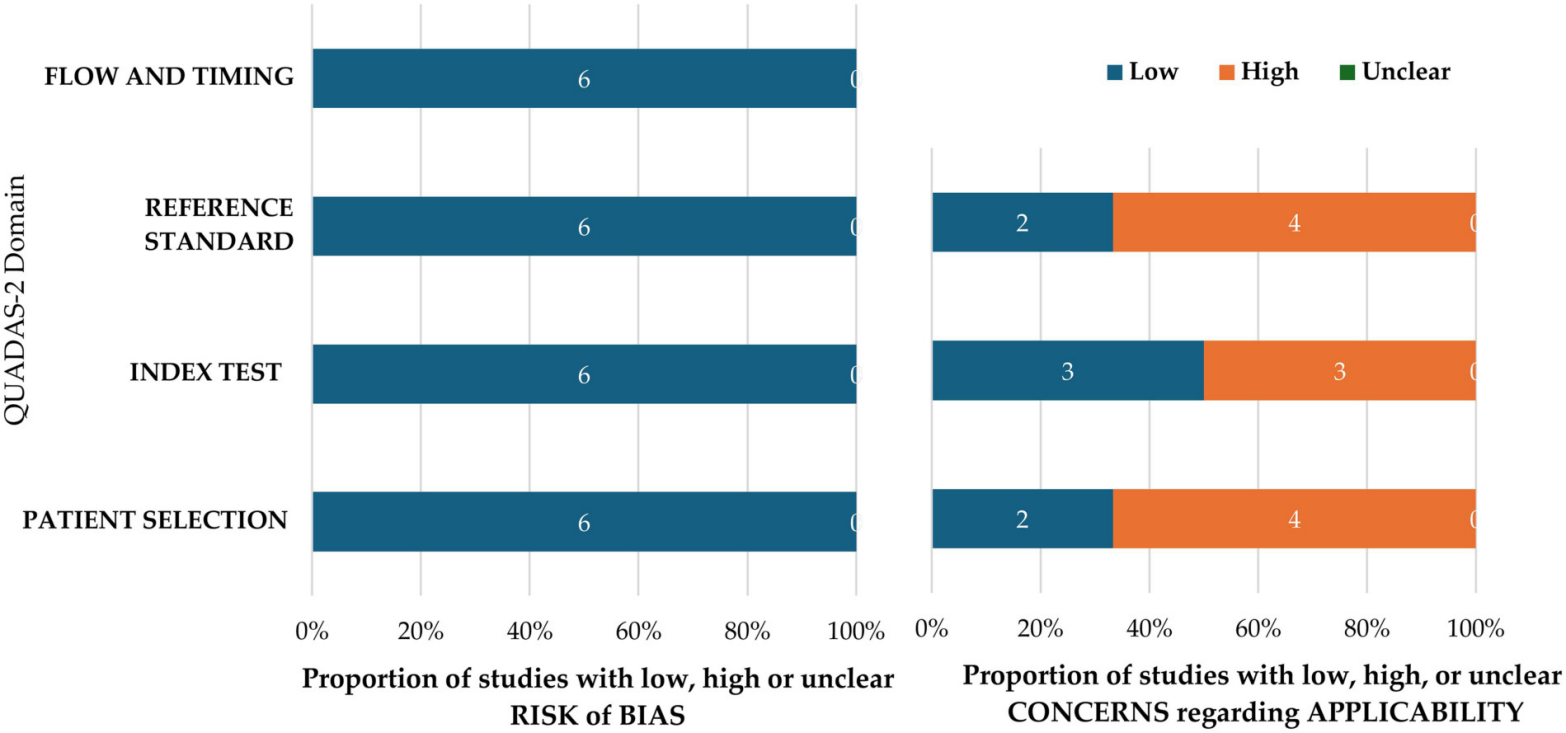
QUADAS-2 assessment of the individual risk of bias domains and applicability concerns.

The QUADAS-2 assessment was used to evaluate methodological quality; however, a clearer distinction between risk of bias (internal validity) and applicability (external validity) is necessary. Risk of bias refers to whether the study design or conduct may have systematically influenced the results, whereas applicability concerns the generalizability of findings to real-world clinical settings. Although several studies demonstrated a low risk of bias in patient selection due to clearly defined criteria and standardized protocols (18–21), many relied on extracted teeth, cadaveric specimens, or *in vitro* datasets rather than clinical patient populations (18, 20, 21). While such designs enhance experimental control, they substantially limit applicability, as they do not capture the variability and complexity of *in vivo* conditions. The absence of external validation across all studies (18–23) further restricts generalizability. Therefore, despite acceptable internal validity in some domains, the overall translational relevance of current AI models for routine clinical practice remains limited. Overall, considering all categories across the included studies, there was a substantial likelihood of high risk of bias in both arms. Further details regarding the QUADAS-2 risk of bias assessment are provided in the (Figure 2; Supplementary Table S3).

### Assessment of strength of evidence

The reliability of the studies featured in this review was assessed utilizing the GRADE approach. The five factors that influence the certainty of evidence include inconsistency, indirectness, imprecision, risk of bias, and publication bias. Evidence can be classified as having very low, low, moderate, or high certainty. Three of the included studies (Saghiri M. A. et al. (18), Saghiri M. A. et al. (19), and Qiao.X. et al. (20), employed relatively small sample sizes, leading to wider confidence intervals and reduced precision. Additionally, four studies (Saghiri M. A. et al. (18), Saghiri M. A. et al. (19), Qiao X. et al. (20), and Thakur V.S. et al. (21), reported a risk of bias, which consequently lowered the overall certainty of the evidence to low (Table 2).

**Table 2:** Assessment of strength of evidence.

Authors	Inconsistency	Indirectness	Imprecision	Risk of bias	Publication bias	Strength of evidence
Saghiri M. A. et al. (18)	Not present	Not present	Present	Present	Not present	⊕⊕◯◯
Saghiri M. A et al. (19)	Not present	Not present	Present	Present	Not present	⊕⊕◯◯
Qiao, X.et al. (20)	Not present	Not present	Present	Present	Not present	⊕⊕◯◯
Thakur, V.S. et al. (21)	Not present	Not present	Not present	Present	Not present	⊕⊕⊕◯
Herbst, S.R. et al. (22)	Not present	Not present	Not present	Not present	Not present	⊕⊕⊕⊕
Latke, V. and Narawade, V. (23)	Not present	Not present	Not present	Not present	Not present	⊕⊕⊕⊕

⊕⊕⊕⊕, high evidence; ⊕⊕⊕◯, moderate evidence.

### Overall summary of the included studies

The AI models showed a sensitivity (0.85–1.00), specificity (0.50–1.00) and accuracy of (0.70–0.95) respectively. The risk of bias was evaluated using the QUADAS-2 assessment tool. Overall, across all categories in the included studies, there was a substantial likelihood of a high risk of bias in both arms. The certainty of the included studies was assessed using the GRADE approach, which indicated that four studies had a risk of bias, consequently lowering the overall certainty of the evidence to low.

In this systematic review, the included studies differ substantially in study design (*in vitro*, cadaveric, and clinical), reference standards, and imaging modalities. Therefore, performing a meta-analysis is inappropriate due to extreme clinical and methodological heterogeneity.

## Discussion

This systematic review examined the application of AI models designed for determining working length during endodontic procedures. Based on qualitative synthesis from six studies published between 2012 and 2024, the findings suggest that AI models have demonstrated promising diagnostic performance, with varying degrees of accuracy and clinical applicability. Despite the heterogeneity of study designs, data sources, and algorithms, a general trend toward enhanced decision-making support via AI integration is evident. Although some of the included studies involved limited sample sizes, they were retained because they reported quantitative diagnostic accuracy outcomes and represented early, methodologically structured applications of AI in endodontic working length–related assessment.

The analysis revealed that various AI algorithms were employed to determine working length; however, due to the qualitative nature of the synthesis and diversity of methodologies, the specific models used were not uniformly reported across studies. Nonetheless, the included studies utilized intraoral radiographs and, in some cases, extracted teeth or cadaveric samples for model development and evaluation. The inclusion of *in vitro* and cadaveric studies was considered appropriate within a diagnostic accuracy framework, as these models are commonly employed in early-phase evaluations of diagnostic technologies. These studies offer controlled conditions with clearly defined reference standards, allowing for precise assessment of algorithmic performance prior to clinical application. However, limitations concerning external validity and generalizability were acknowledged and addressed in the QUADAS-2 applicability assessment and GRADE certainty ratings. Most studies assessed performance using metrics such as accuracy, with results indicating that AI systems have the potential to support or approximate expert-level performance in identifying endodontic working lengths.

These findings align with previous literature suggesting the utility of AI in dental diagnostics. Systematic reviews have highlighted the potential of AI in detecting periapical lesions and have reported that AI models demonstrate high specificity and sensitivity in identifying periapical radiolucencies (11, 22). Previous literature has demonstrated that AI-based diagnostic tools can effectively reduce inter-observer variability in endodontic measurements, reinforcing the conclusions drawn from the present analysis (14, 24).

However, including *in vitro* and cadaveric studies presents an inherent limitation, as results obtained in controlled experimental environments may not accurately reflect real clinical situations. Additionally, several methodological issues must be acknowledged. A key limitation across the studies is the variability in datasets, with some using extracted teeth and cadaveric samples instead of data from actual patients. This raises concerns about ecological validity and the generalizability of the findings. Furthermore, Qiao X. et al. (20) demonstrated unclear reporting regarding the reference standards and timing of index tests, resulting in an uncertain risk of bias across multiple QUADAS-2 domains. These points underscore the necessity for standardized protocols in AI research, particularly concerning dataset quality, model training, and transparent validation processes. A common limitation identified in the studies was the lack of detailed technical information regarding AI model design, feature engineering, and how datasets were divided. None of the studies performed external validation, which limits the generalizability of the findings.

The reported ranges of sensitivity, specificity, and accuracy should be interpreted cautiously due to significant variations in outcome definitions, reference standards, and study designs among the included research (18–23). In these studies, what constituted a “positive” result varied widely, including correct localization of the apical foramen (18), determination of working length (19, 23), measurement of root canal length using impedance-based techniques (20), classification of apical extent (21), and prediction of optimal root filling length (22), which limits direct comparison. Although accuracy was often reported as the primary performance measure (18–23), it can be misleading when datasets are imbalanced and does not fully capture diagnostic discrimination. Sensitivity and specificity were reported inconsistently (22), and confidence intervals were rarely provided (18–23), restricting the evaluation of statistical reliability and uncertainty. Notably, none of the studies explicitly defined clinically acceptable error margins for working length determination (such as ±0.5 mm or ±1.0 mm compared to the reference standard), despite the importance of these thresholds in clinical endodontics (18–23). Additionally, the reference standards varied widely, including expert endodontist evaluations (18, 19), dual-frequency impedance ratio methods (20), radiographic classification systems (21), and radiographic outcome-based definitions (22, 23), further limiting cross-study comparisons. Therefore, the reported performance metrics should be considered specific to each study rather than directly comparable, and any conclusions regarding the superiority of one model over another should be made cautiously given these methodological differences (18–23).

Despite these limitations, the potential clinical implications of AI in endodontics are substantial. Accurate determination of the working length is critical for procedural success, minimizing complications such as underfilling or over-instrumentation (25–27). AI could serve as a reliable adjunct, especially in complex anatomical cases or where practitioner experience varies. Moreover, AI systems could enhance efficiency in busy clinical settings by providing real-time, reproducible measurements that reduce diagnostic uncertainty.

Future research should prioritize the use of large, annotated clinical datasets, preferably multi-center in origin, to train and validate AI models. The development of consensus guidelines for AI deployment in endodontic imaging is also warranted. Additionally, longitudinal clinical trials are needed to evaluate whether AI-assisted diagnostics improve long-term treatment outcomes compared to conventional techniques.

## Conclusions

Although several studies have reported high diagnostic performance, none have provided high-certainty evidence from prospective, real-world clinical trials. Most investigations were retrospective, *in vitro*, or based on internally validated datasets without external validation. Consequently, the current evidence base remains preliminary. While promising, current applications are limited by methodological variability and require further validation before widespread clinical adoption. Prospective studies and randomized clinical trials are essential for evaluating the effectiveness and cost-efficiency of deep learning-based lesion detection in real clinical settings. Future research should focus on standardizing test application and reporting results in more diverse and well-defined patient populations to achieve a more precise and generalizable estimate of diagnostic performance. Continued interdisciplinary collaboration among dental clinicians, radiologists, and data scientists is essential for integrating these technologies into routine practice.

## Data Availability

The original contributions presented in the study are included in the article/Supplementary Material, further inquiries can be directed to the corresponding author.
